# Troponina-T e Peptídeo Natriurético tipo B na COVID-19

**DOI:** 10.36660/abc.20201191

**Published:** 2021-04-08

**Authors:** Sora Yasri, Viroj Wiwanitkit

**Affiliations:** 1 Dr. DY Patil University Pune Índia Dr. DY Patil University, Pune - Índia

**Keywords:** COVID-19/complicações, Betacoronavírus, Mortalidade, Hospitalização, Miocardite, Biomarcadores, Troponina T, Peptídeo Natriurético Tipo B

Caro Editor,

Gostaríamos de compartilhar ideias sobre a publicação “Valor Prognóstico da Troponina T e do Peptídeo Natriurético Tipo B em Pacientes Internados por COVID-19.^1^ Almeida Jr. et al.,^1^ relataram o uso da caixa de memória como uma ferramenta para apoiar o luto e concluíram que “Nas primeiras 24h de admissão, TnT, mas não o BNP, foi marcador independente de mortalidade ou necessidade de ventilação mecânica invasiva.”^1^ Na COVID-19, a complicação cardíaca é possível e comum em infecções graves.^2^ O comprometimento miocárdico é um problema cardíaco comum e a miocardite imunológica é uma importante manifestação grave da COVID-19.^3^ Portanto, a troponina-T, que é um bom biomarcador de lesão miocárdica, pode ter um bom fator prognóstico para a gravidade da COVID-19. No entanto, deve-se notar que problemas renais também podem induzir alterações da troponina-T^4^ Se o paciente tiver um problema renal subjacente, a interpretação da troponina-T em pacientes com COVID-19 deve ser cuidadosa.
