# Supplementary material for: Troponina-T e Peptídeo Natriurético tipo B na COVID-19
Source: Arq Bras Cardiol. 2021 Apr 8;116(4):854. [Article in Portuguese] doi: 10.36660/abc.20201191 (PMC8121388; doi:10.36660/abc.20201191)
Supplement: Translation

# Translation: Troponin-T and B-Type Natriuretic Peptide in COVID-19

**Authors:** Sora Yasri, Viroj Wiwanitkit

**Affiliations:** 1 Pune India Dr. DY Patil University, Pune - India

**Keywords:** COVID-19/complications, Betacoronavirus, Mortality, Hospitalization, Myocarditis, Biomarkers, Troponi-T, Natriuretic B-Type

Dear Editor,

We would like to share ideas on the publication “Prognostic Value of Troponin-T and B-Type Natriuretic Peptide in Patients Hospitalized for COVID-19 [1].” Almeida Jr. et al.^1^ reported using Memory box as a tool to support grief and concluded that “*In the first 24h of admission, TnT, but not BNP, was an independent marker of mortality or need for invasive mechanical ventilation.*”^1^ In COVID-19, cardiac complication is possible and is common in severe infection.^2^ Myocardial involvement is a common cardiac problem and immunological myocarditis is an important severe manifestation of COVID-19.^3^ Therefore, troponin-T, which is a good biomarker for myocardial injury, might have good prognostic factor for severity of COVID-19. Nevertheless, it should be noted that renal problems can also induce troponin-T abnormality.^4^ If the patient has an underlying renal problem, the interpretation of troponin-T in COVID-19 patients must be careful.
